# The cardiac pacemakers: A paradigm of robustness in evolutionary biology

**DOI:** 10.1113/JP287139

**Published:** 2025-09-05

**Authors:** Denis Noble

**Affiliations:** ^1^ Department of Physiology, Anatomy & Genetics University of Oxford and Daegu‐Gyeongbuk Institute of Science and Technology South Korea

**Keywords:** cardiac pacemakers, causation in physiological networks, central dogma, difference between association and causation, DNA self‐replication, genome‐wide association studies

## Abstract

The cardiac pacemaker activity is formed from multiple interlocking physiological networks, any one of which can generate rhythm. The interlocking is reciprocal so that they automatically replace each other. In such interlocking control systems, the association scores for individual components are necessarily low, even though causation, measured by the electric current carried by the relevant ion channels, is large. This kind of reciprocally based robustness is widespread in living organisms, which explains why most association scores in genome‐wide association studies are low, or even zero. It also explains why the polygenic scores do not reliably predict disease states. Genomics alone is therefore a poor method for the discovery of treatments for polygenic diseases. Reliance on genomics has led to a gene‐centric impasse in healthcare, which requires a shift in favour of physiological studies that can reveal genuine causation rather than just association. The case for such a shift includes understanding that DNA is not a good self‐replicator in very large genomes. Self‐replication of DNA and RNA in purely chemical environments confirms that fact. The error rate would amount to hundreds of thousands in a genome of three billion base pairs. Living cells can orchestrate the enzymes necessary for nearly perfect replication before cell division by correcting those errors. These cellular proof‐reading processes also open the way to control processes that are used to generate new DNA sequences when the immune and other systems need to do so.

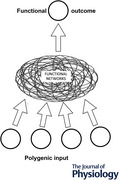

## Graphical abstract

**Abstract figure legend** Functional networks in living systems are formed by many thousands of gene products. In association with those networks, several genes (four in this diagram) may be sufficient, each on its own, to ensure that the function occurs. Any one of these may be removed or blocked while leaving the others to continue functioning. Each will then display a low, or even zero, association score in genome‐wide association studies. In such polygenic interlocking systems, causation will then be hidden beneath the buffering properties of the networks. Measuring causation may then be revealed in physiological models of the networks. By revealing causation, we may then hope to discover cures for multi‐factorial diseases.

## Introduction

This short review article will explain what robustness in functional physiological control networks means, and what are its developmental functional and evolutionary implications. I will use pacemaker rhythm in the heart as my paradigm example, since that is the one whose robustness I understand best. It was first discovered, both experimentally and in detailed mathematical modelling, over 30 years ago (Noble et al., 1992).

I will also explain how we now know that this kind of robustness is typical of many other physiological functional networks, probably over 90%. The exceptions are the monogenetic diseases affecting around 4–5% of the population.

I will then explain why robustness leads to ways in which physiology can distinguish between association and causation. That is the key to whether it can guide us to the research that may lead to therapies for polygenic diseases. Significantly, that is precisely what happened in the case of heart rhythm, where the useful rhythm‐slowing medication, ivabradine (Procoralan), was the clinical outcome. Studying functional higher‐level networks (see Fig. 2 below) could, in principle, lead to other successful medications arising naturally from physiological understanding of causation in health and disease.

I will then explain why the current standard theories of the fundamentals of biology, and of evolutionary biology in particular, have supported the wrong strategies in clinically relevant medical research.

Finally, I will outline a pathway for guiding medical research out of what I call the ‘gene‐centric impasse’, which is urgently needed (Baverstock, 2024; Noble, 2024a; Noble & Noble 2025).

### What do genes do? The standard view

Commonly held views of genes frequently confuse association with causation. In a debate with me three years ago, Richard Dawkins began by citing my book, *Dance to the Tune of Life*:

‘This book will show you that there are no genes “for” anything. Living organisms have functions which use genes to make the molecules they need. They are not active causes.’ (Noble, 2016, p. x).

In his recent book, *The Genetic Book of the Dead*, he explained why he did not then, and still does not now, accept that statement. He wrote:

‘Successful genes are those with a statistical tendency to inhabit bodies that are good at surviving and reproducing. And they enjoy that statistical tendency, positive or negative, by virtue of the causal influence they exert over bodies. So we have arrived at the reason why it was profoundly wrong to say that genes are not active causes. Active causes is precisely and indispensably what they must be. If they were not, there would be no natural selection and no adaptive evolution.’ (Dawkins, 2024, p. 183).

This quote reveals a misuse of the term ‘active causes’. Such causes are precisely what we should be seeking in order to develop effective cures for diseases, but DNA sequences cannot be active in the relevant sense. They are sequences (a form) whose order can be read by living cells. But as a chemical, DNA is inert. As I will show in this article, physiology can unravel the passive and active forms of causation. Genome sequencing alone cannot achieve this. That conclusion is obvious when we consider that active causation can occur even when the association score is negligible or zero.

Yet, in an unfortunate failure to appreciate this difference between passive and active causation in biology, the United States leader of the Human Genome Project, Francis Collins, declared in 1999, a year or two before the publication of the first human genome sequencing:

‘The history of biology was forever altered a decade ago by the bold decision to launch a research program that would characterize in ultimate detail the complete set of genetic instructions of the human being… This knowledge will dramatically accelerate the development of new strategies for the diagnosis, prevention, and treatment of disease, not just for single‐gene disorders but for the host of more common complex diseases (e.g. diabetes, heart disease, schizophrenia, and cancer) for which genetic differences may contribute to the risk of contracting the disease and the response to particular therapies.’ (Collins, 1999).

That lecture was delivered 26 years ago, and included the prediction that, within 10 years of sequencing the full human genome, ‘diagnosis, prevention and treatment of disease’ would be the outcome. Collins even designed an imagined ‘Case Report’ for the year 2010 (see Collins, 1999, Table 1), imagined as leading to successful treatment for cancer. A quarter of a century later, almost none of this has happened *for any of the specified diseases*. Why?

As physiologists, we know why. Association, which is essentially all that genomic sequencing can tell us, *is not the same as causation*. And, if we do not know what causes a disease, we have little chance of finding a cure. How do we study causation? That is precisely what I will explain in a later section.

Yet, for the whole duration of that quarter of a century what we have witnessed in the UK is the closure by many universities of one physiology department after another. The funding dried up.

How did we end up in this situation in which the field that has a proven track record during the 20th century of discovering useful medications, through investigation of functional levels of organisation, has been sidelined so comprehensively?

I move on to the phenomenal discoveries that motivated the standard view and led to that situation: the central dogma of Francis Crick. I will show how those undoubtedly important discoveries were nevertheless misinterpreted to imply that physiology had become irrelevant.

### Crick's ‘central dogma’ view

The work of Watson and Crick in Cambridge, and Wilkins and Franklin in London, led to one of the most important discoveries in molecular biology: that DNA exists in living cells as a double helix (Watson & Crick 1953). The Quantum Mechanics pioneer, Schrödinger, had already deduced (Schrödinger, 1944) that genetic information in organisms would need to be at the molecular level if the huge amount of information needed was to be contained and transmitted to subsequent generations. Since he also thought that this molecular information needed to be sufficient to fully characterise the organism, he also proposed a process by which it could be accurately copied and transmitted from one generation to the next.

At that time, biologists interested in molecular structure were already using X‐ray crystallography to determine the structure of complex molecules of biological importance. Dorothy Hodgkin had determined the structures of cholesterol in 1937, and was soon to do so also for penicillin and vitamin B12. Her experiments were performed on crystals. It was natural therefore for Schrödinger to propose that the genetic material would need to be crystalline to reproduce *itself* in the way ‘how crystals are formed’. But he also knew that such a ‘crystal’ would need to contain a lot of information. Most crystals do not contain more repetitive (periodic) structural information than that which determines which other molecules can join the crystal. A biological crystal responsible for inheritance would, by contrast, need to be what he called aperiodic. By that he meant that it would be information‐dense, not just repeating structural sequences as in normal crystals, which only grow; they do not have to transmit vast amounts of information to a new generation. The discovery of DNA structure as a double helix reinforced Schrödinger's view, since it then became easy to imagine that, once unwound, the two threads could attract the complementary nucleotides to bind to it, A and T together, C and G together. They could then automatically reform the double helix. It seemed therefore that the replication process was solved.

**Figure 1:**
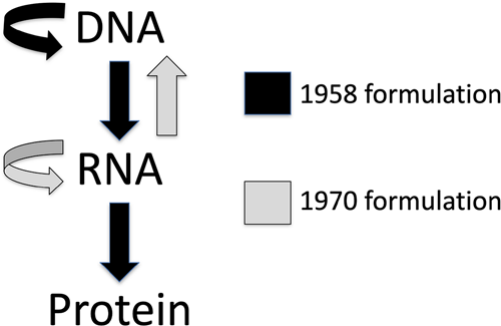
The Central Dogma of Molecular Biology, as redrawn by Crick in 1970 following the discovery of back‐transcription from RNA to DNA There is no back‐transcription possible between proteins and nucleotide sequences. Note also that the self‐replication arrows are solid, implying accurate self‐replication (Crick, 1970). Figure 2 will show the correct interpretation as dotted arrows, implying only weak self‐replication.

Crick (1958) formulated the process by which proteins are formed as a determinate three stage process: DNA ‐> RNA ‐> PROTEIN. When back‐transcription of RNA to DNA was discovered, he modified this diagram in 1970 to become the now standard version of his ‘central dogma’ (Fig. 1).

Notice that there is no backward arrow from Protein to RNA or DNA. This represents the fact that there is no process by which living cells could achieve it. Yet, something must nevertheless be missing from this diagram since organisms under stress, such as the arrival of a new virus or bacterium, or metabolic stress, are clearly capable of reorganising their DNA sequences. The immune system can generate vast numbers of new immunoglobulins when it needs to, while bacteria can rapidly evolve their DNA when under stress. Barbara McClintock clearly demonstrated this process in maize plants for which she was awarded the 1983 Nobel Prize for Physiology and Medicine (McClintock 1984), Shapiro did the same for bacteria (Shapiro, 1983, 2011, 2022), while the ability of the immune system to mutate the variable part of the coding for immunoglobulins in a targeted process is also well‐established (Odegard & Schatz, 2006).

Something very fundamental must therefore be missing from Crick's diagram in Fig. 1.

### The view from chemistry

The view from chemistry can provide a large part of the answer. For the nucleotide strings to be (nearly) perfect *self*‐replicators with no input from the living cell they should be able to achieve this when unwound in a purely chemical environment. Deck et al. (2011) did this for RNA, while Schulman et al. (2012) did the experiments for DNA. In both cases, self‐replication happened, and its accuracy is impressive. Only one error in a few thousands of base pairs. The titles of their articles even indicate that. Schulman et al. refer to ‘*robust* self‐replication’ while Deck et al. refer to ‘*efficient* enzyme‐free copying’ (my emphases).

And, indeed, this accuracy of self‐replication works for relatively small viruses with just a few thousand base pairs.

But, in a genome of three billion base pairs, that process would leave hundreds of thousands of errors. That degree of error would be lethal. With a genome of three billion base pairs, the daughter cells could hardly function at all.

That fact is the Trojan horse in my story. Since copying of DNA is in fact nearly perfect in each division to form two daughter cells, living cells must possess the means to achieve such accuracy. How do they do that?

### Physiological view

Living cells do indeed correct virtually all of those errors, and they do not divide until that process is complete. Cells with heavily damaged DNA are destroyed. To achieve this, the control networks orchestrate a number of cut‐and‐paste enzymes to walk along the DNA threads, discover the errors, correct them, and continue to creep and correct, working along the threads until an error rate of only one in 10 billion becomes possible.

Two important physiological conclusions can be drawn from these facts.

First, in large genomes, DNA is not a nearly perfect self‐replicator outside a living cell. In genomes larger that a few tens of thousands of base pairs, the result would be disastrous.

Second, since the cut‐and‐paste enzyme processes are under the control of the living cell, that control can be varied. Although the details vary according to the processes involved, the ability of the immune system, and of other systems of the body, to produce new DNA sequences is no longer a mystery. This fact is also important. If DNA did indeed accurately self‐replicate independent of any control by the cell, that ability of the immune system could not even exist. The system needs to harness the stochasticity created by the errors of purely chemical self‐replication.

**Figure 2:**
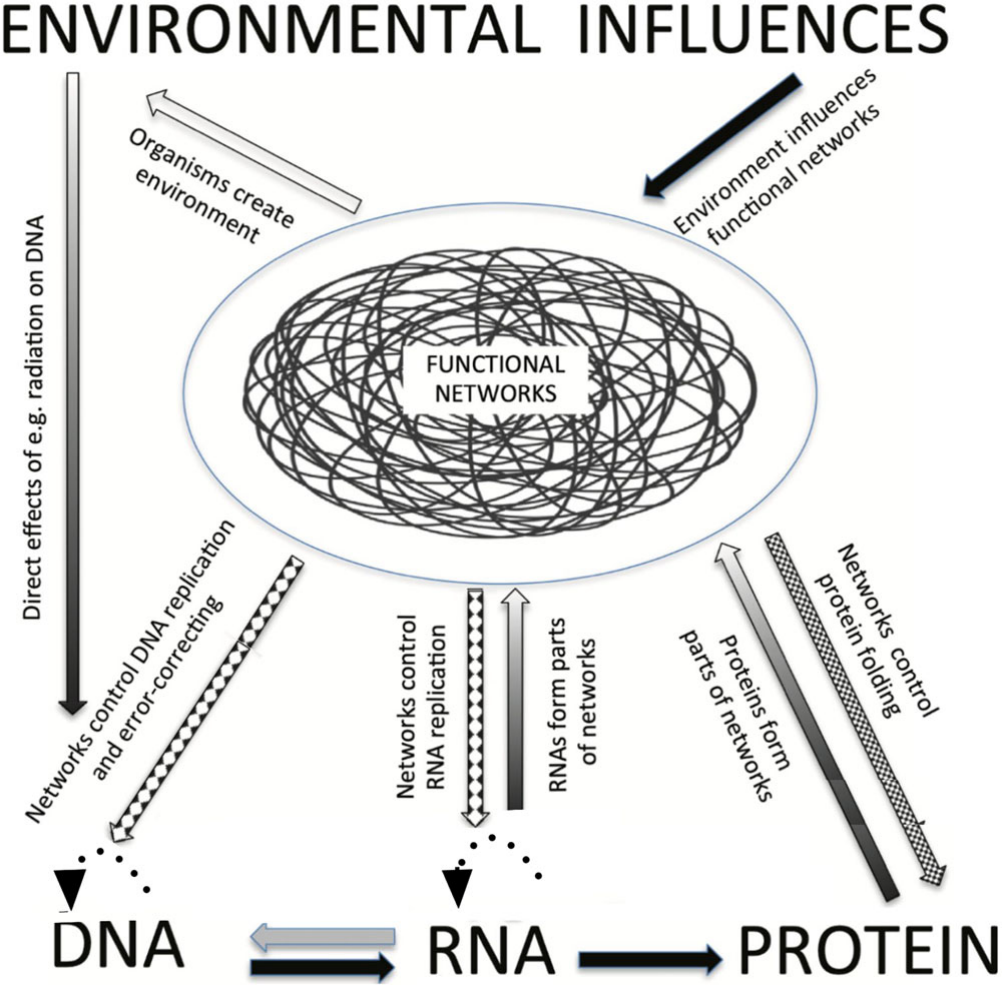
The role of physiological functional networks in the control of DNA, RNAs and proteins Slightly redrawn from Noble & Noble (2025, Fig. 1). The dotted self‐replication arrows have been placed above the nucleotides instead of below. The downward control arrows are then correctly placed in relation to self‐replication. Note also that we call the networks ‘functional’ rather than using the common term ‘gene regulatory networks’. That term is often misunderstood to imply that genes are doing the regulation.

Figure 2 is taken from a recent paper with my brother Raymond Noble, published in the journal *Evolutionary Biology* in June 2025 (Noble & Noble, 2025). I have slightly redrawn the diagram by placing the back‐curved dotted arrows on top of the DNA and RNA instead of underneath. That diagram makes two important facts clear. First, those arrows are thinly dotted because, as already explained, self‐replication is very weak in genomes as large as three billion base pairs. Therefore, in long genomes, DNA cannot self‐replicate ‘how crystals are formed’. Second, placing the arrows above the nucleotide sequences makes it evident that they are both under control by the physiological networks orchestrated by the living cell. That is how accurate transmission of DNA to subsequent generations of daughter cells, and to subsequent generations of organisms, becomes possible.

It is important to understand the role of the other arrows in this figure in order to understand how organisms embody robustness in their physiological functions.

There are two upward arrows from the ‘central dogma’ part of the diagram. The first contributes many RNAs to the physiological networks. Unlike DNA, RNAs can act as enzymes, but not as well as proteins can, and their main role in organisms is to act as control signals to DNA. The second upward arrow from the ‘central dogma’ to the networks represents the contribution of proteins to the networks where they carry out their functions. There is also a downward arrow from the networks to the proteins since, as Ball (2024) documents so fully, most proteins (around 70%) do not have a fixed three‐dimensional structure. Protein folding, and therefore the function of each protein, is under network control. Many have multiple functions. The existence of causal arrows in both directions, up and down, reflects the Principle of Biological Relativity, i.e. the statement that ‘there is no privileged level of causality’ (Noble, 2012, 2016, p 169).

The proteins, RNAs, metabolic products, hormones, transmitters, the structural features of intracellular organelles, the electrical, and perhaps also magnetic, fields of cells and organelles, form networks of almost incalculable complexity. This is where organisms generate their robustness.

Robustness cannot therefore be a property of individual genes (protein‐coding DNA) and proteins. They contribute to robustness only by being part of the functional physiological networks. Physiology is precisely the study of those networks, which is why it will be the key to discovering treatments for polygenic diseases which cannot readily yield to individual gene therapy.

This section of my review places the ‘central dogma’ in its relevant perspective. It becomes clear that it is neither central nor a dogma. It is simply an important chemical fact about the way in which protein sequences are formed. It was hyped up to be more than that and has misled biology for nearly 70 years (Noble & Noble, 2023). It does not help in understanding accurate self‐replication of DNA since it omits the processes orchestrated by living cells that make highly accurate replication possible.

### The cardiac pacemaker as a paradigm of robustness

I was a doctoral thesis student working at University College London under Otto Hutter in 1960. The origin of rhythmic electrical activity in the heart was still unexplained. Draper & Weidmann (1951); Weidmann (1956) produced the first intracellular recordings of cardiac action potentials from Purkinje fibres of the ventricle which, although not the heart's natural pacemaker, the sinus node, nevertheless displayed spontaneous depolarisations leading to rhythmic activity. Hutter and I identified two potassium channels generating electric current in Purkinje fibres (Hall Hutter & Noble, 1963; Hutter & Noble, 1960). I proposed to my supervisor to formulate equations for these channels, combine them with the sodium channel from the Hodgkin‐Huxley (1952) nerve equations to see whether the combination of equations could generate the rhythm observed experimentally in Purkinje fibres.

The detailed story of how that led to my 1960 and 1962 papers (Noble, 1960, 1962) is explained in a book, *The Pacemaker Channels of the Heart, From Reductionism to Systems Biology*, now in Press (Noble, 2025) to be published this autumn. The idea worked. The model generated electrical changes, including the rhythm, very similar to what Weidmann had recorded experimentally. It also explained many other features of his experiments, including impedance changes during activity, all‐or‐nothing repolarisation, and the influence of potassium ions on the action potential duration, some of which were counter‐intuitive.

Although I did not notice the fact at the time, the model had a serious problem. If anything damaging happened, such as a critical genetic mutation, or a block of any of the channels by a drug, the rhythm would immediately cease. Even though highly successful in explaining many otherwise puzzling experimental results, the model was therefore fragile.

Fast‐forward three decades to 1992. By then my laboratory at Oxford had passed through several major revisions of the original pacemaking model Noble (1984) and, as a result, could tell a different, more nuanced, story (Noble et al., 1992). In fact, the pacemaker processes (note the plural) of the heart are extremely robust. At least three networks within the functional physiological networks could ensure that rhythm would continue if any of the others became inoperable for genetic mutation or drug treatment reasons. What we published in 1992 is therefore extremely robust.

The full details of that 30‐year development will also be found in Noble (2025). It will suffice for this review article to highlight the key facts and discoveries. What were the differences between what was done in 1960/1962 and what was published in 1992?

Although my laboratory had continued to work on the Purkinje fibres, we had also branched out to study the sinus node. In our modelling work to complement the experimental findings, we had incorporated many more ionic channels, ion exchange transporters, intracellular calcium signalling processes, and much else relevant to generating rhythm. We had also obtained the relevant experimental data for the modelling parameters from single‐cell experiments on sinus node cells recorded under patch‐clamp conditions. None of these experimental techniques were available in 1960.

**Figure 3:**
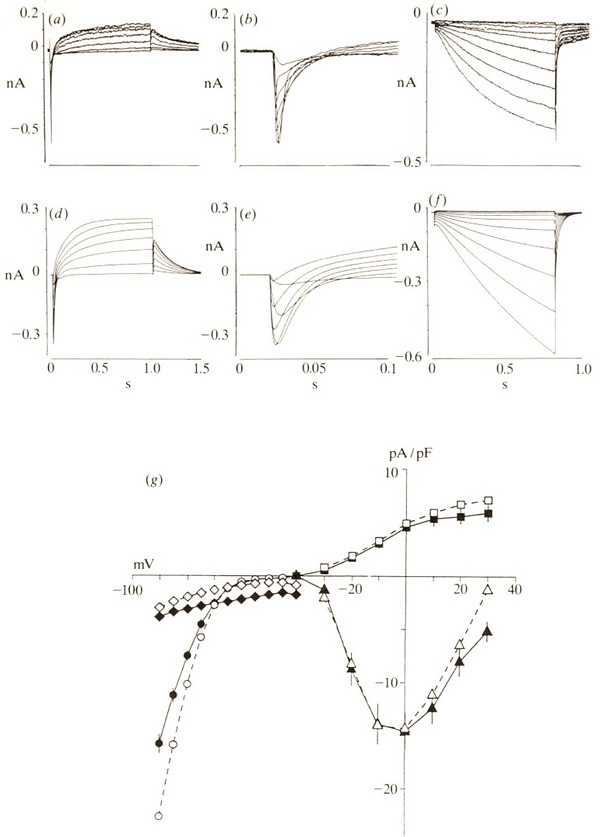
Experimental ionic current traces and current‐voltage graphs from a single sinus noide cell *A–C*, examples of experimentally recorded currents under voltage‐clamp conditions in a single rabbit sinus node cell. *D*–*F*, computed currents obtained under voltage‐clamp conditions using the parameters fitting the mean currents as in *G*. *A*, a cell was held at ‐40 mV and given depolarising pulses to +30 mV in successive 10 mV steps of 1 s duration at a frequency of 0.2 Hz. Current traces are shown superimposed. *B*, first 100 ms of currents in *A*, shown on an expanded timescale, (*C*) current records obtained during hyperpolarising pulses. The cell was held at ‐40 mV and given successive ‐5 mV steps of 800 ms duration down to ‐90 mV at a frequency of 0.2 Hz. *D*–*F*, computer simulations using the same protocols as in *A*, *B* and *C*, respectively. *G*, current density–voltage relations for the experimentally recorded (solid symbols) and computed (open symbols) currents shown in *A–C*. The peak *I*_K_ tail current (squares) and the peak inward calcium current (triangles) measured with respect to the holding current; i_f_ (circles) was measured as the difference between current level attained upon hyperpolarisation and the current at 800 ms; z'b (diamonds; net background current which includes i_bNa_) was measured with respect to the zero current level.

The quality of the experimental basis of the model revealing the robustness can be judged from the data in Fig 3.

The legend of Fig. 3 includes all the relevant details given in the 1992 paper. Readers interested in the full details are referred to the paper itself, and to Noble (2025). The essential message for this review is the accuracy with which the single sinus node cell was represented by the fine‐tuning of the equations. We performed these experiments and simulations precisely because we wanted the modelling outcome to be as experimentally reliable as possible.

**Figure 4:**
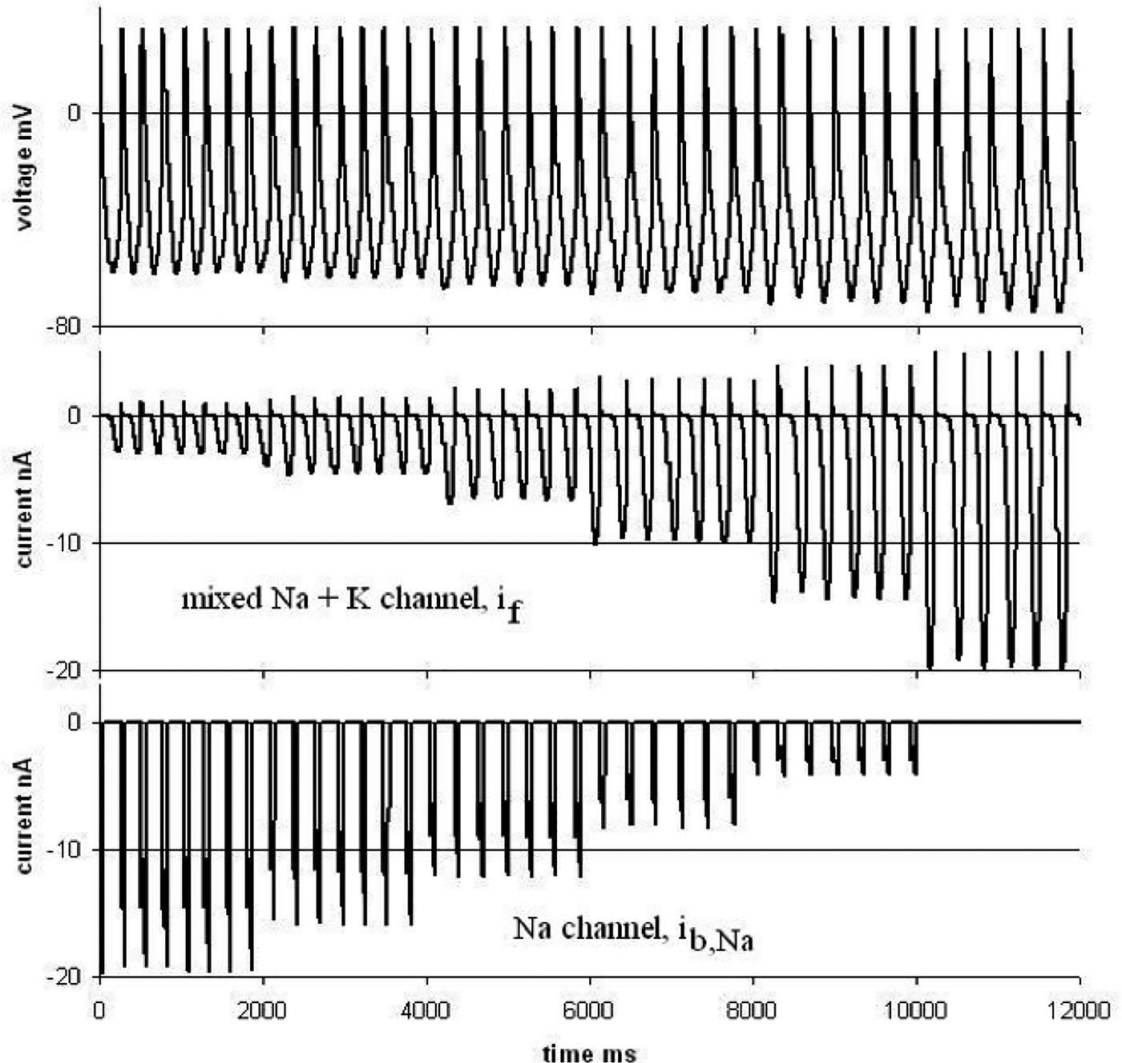
Simulation of voltage changes (top) and ionic current changes (middle and bottom) during stepwise block of background sodium channel *Top*, membrane potential variations as the background sodium channel (bottom plot) is progressively reduced from 100% normal value to 80, 60, 40, 20 and 0%. The result is a very modest fall in frequency. *Middle*, the reason is that as the i_b,Na_ channel is reduced, the i_f_ channel activation is increased, almost in proportion to the reduction of the other channel (From Noble, 2025, Fig. 6.2).

Figure 4 shows the results of a simulation of robustness by the model.

The relative proportions of the different ionic currents capable of sustaining rhythm varies depending on where cells are situated within the sinus node. The central cells are relatively depolarised compared with the peripheral cells. As the background channel is reduced, the model cell becomes increasingly hyperpolarised, so moving towards the characteristics of a peripheral cell, where i_f_ dominates as the rhythm generator. The hyperpolarisation is responsible for activating the i_f_ channel to an increasing degree. But the change in frequency is only around 10–15%.

In addition to the two mechanisms evident here, there is also an intracellular calcium oscillator capable of activating depolarising current via the sodium–calcium exchanger (Lakatta et al., 2010) and, as we already know, the Purkinje fibre system can also generate rhythm, albeit at a lower frequency than the sinoatrial node. The heart as a whole therefore has around four processes by which rhythm can be maintained.

What has been discovered therefore is that increased complexity of the functional physiological networks involved in heart rhythm has allowed evolution to generate powerful back‐up processes that can readily replace each other. Figure 4 demonstrates the i_f_ system replacing a background depolarising current, but the reverse also applies. Following the discovery of i_f_ by Dario DiFrancesco in 1980, and its incorporation into the Oxford group's computer models, he was awarded the Grand Prix LeFoulon‐Delalande by the Academie des Sciences in Paris in 2008. By then the French pharmaceutical company, Servier, had developed the medication ivabradine, a specific blocker of this channel. Using ivabradine to block i_f_ also produces only a modest 10–15% reduction in frequency, whereas its contribution to the current causing the pacemaker depolarisation can be anywhere up to 85% depending on the region of the sinus node and the concentration of adrenaline. It is the ability to reveal causation that distinguishes physiology from association studies, and why such physiological understanding of causation is a prerequisite for successful treatments.

In addition to these ion channel mechanisms of redundancy that back‐up sinus node rhythm, there are further protections of rhythm in the whole heart, arising from structural redundancies, resistance to slowing by acetylcholine, latent pacemakers, biochemical drivers (e.g. compartmentalised cAMP) and stretch‐activated processes (Kohl et al., 1999; Li, et al., 2017; Ming et al., 2024; Parameswaran, R., et al. 2020; Tomek & Zaccolo, 2023). These modulating processes are important, but they do not so easily allow a quantitative measure of the difference between association and causation. The reason I have focused in this article on the ion channel processes in the sinus node and Purkinje cells is that, through *existing mathematical modelling*, the distinction can readily be quantified. Models that can be used in this way require representation of single‐gene products, such as RNAs and proteins. Otherwise calculation of an association score with particular genes would not be possible.

### Association not causation

There is a further conclusion to be drawn from these experiments and the associated modelling work. They show that an association score, which is the percentage change in function or probability of occurrence of, e.g. a disease state, can be very different from the percentage causation. In the case of the ion channel networks in the heart, the two can be as different as 15% association and 85% causation. Also important is the fact that it can also be stated the other way round. Even a zero association score can hide a large causal effect. It depends on how strong the genetic buffering effect is, i.e. how robust the back‐up redundancy is. Chapter 8 of Noble (2025) is entirely devoted to this question.

When I retired from my Cardiovascular chair at Oxford in 2004, I began searching for other studies showing the same phenomenon. I did not have to wait long to find that what my group had discovered for the heart occurs all over biology.

I soon found the study of Hillenmeyer et al. (2008) on yeast. Yeast has about 6000 genes (DNA sequences coding for proteins). Under ideal physiological conditions, 80% of these can be knocked out with little or no change in function. Yet, most of those genes are demonstrably causal. When yeast is exposed to challenging nutritional environments, most of the knock‐outs that were silent under ideal conditions now showed strong effects. These experiments show that even a zero association score can hide a strong causal effect, which is a further way of demonstrating that association and causation are not the same.

This is precisely what has emerged from extensive measurements of association scores over the 25 years during which human genome sequencing has been done and documented together with the relevant clinical data on which diseases the subjects developed. These data were put to a critical test by Hingorani et al. (2023). They asked the question: how good are the polygenic scores in the polygenic score repository at predicting which diseases the subjects may suffer. The conclusion of that article is clear:

‘Polygenic risk scores performed poorly in population screening, individual risk prediction, and population risk stratification. Strong claims about the effect of polygenic risk scores on healthcare seem to be disproportionate to their performance.’ (Hingorani et al., 2023, p. 1).

Genome‐wide association studies alone can therefore be a very misleading guide to causation. Moreover, since most association scores are unexpectedly low, we can now understand why. Robustness in living organisms is widespread. Causation is massively concealed beneath it, which is why association scores are so far from being equivalent to causation.

The problem this creates is severe. To develop therapies, we need to know what *causes* a disease not just what is associated with a disease. Even a zero association score can hide substantial causation. This outcome is what I call the gene‐centric impasse. Finding our way out of it is now urgent. The multi‐factorial diseases of old age, and the daunting financial burdens on health services (Scott, 2025; Scott & Gratton, 2021), have caught up with us and we still have no cures for the common fatal diseases of old age even from massive genomic sequencing.

### The way forward on designing therapies

In 2020, Peter Hunter and I wrote an editorial for the journal *Epigenetics* (Noble & Hunter, 2020) in which we outlined how physiological modelling could reveal the hidden causation. Since at least 90% of association scores suffer from this problem, it is important to discover causation rather than just association if medical research is to be useful in developing new medications. The way in which physiology achieves that is to study causation within complex networks and derive the relevant differential equations, so that from the solutions we can work out the causal influence of the component processes.

There is a continually growing number of models of complex functional networks in the CellML repository. As those models develop towards representing alternative means by which functionality has been made robust during evolution, it becomes increasingly possible to disentangle causation from mere association. That is why I describe the heart rhythm modelling work as a paradigm. Once we have revealed causation, the way becomes open to discover treatments.

### When were the alarm bells ringing?

Could the problems with polygenic association scores have been avoided? This is an important question. A failure to deliver promised health benefits on the scale of the Human Genome Project is not trivial. It can affect confidence in science itself when the public benefits of a project are over‐hyped.

I was deeply involved in these issues of public confidence in science as one of the founders of the Campaign for Science and Engineering (CaSE – founded in 1986 as Save British Science). Confidence was essential as that organisation moved towards advising the future government of the UK for the decade from 1997 to 2007, when public science funding was largely restored over the period. I was, at the same time, deeply involved (as chair) of the International Congress of Physiological Sciences held in Glasgow in 1993. For that Congress we published and widely distributed a book, *The Logic of Life, the challenge of integrative physiology* (Boyd & Noble, 1993). It is sufficient to cite some of the relevant chapters of that book:

‘Perhaps it would be better therefore to picture genes as severely restricted prisoners rather than as free‐roving “selfish” entities: prisoners of the successful physiological systems carrying them.’ (Noble & Boyd, 1993, p. 5).

‘The triplet code is only machine language… The programme resides at a higher level of control and regulation – and we know virtually nothing about it.’ (Gould, 1993, p. 19).

‘Every “level” in a natural system is constrained by the next level below and the next level above; it is in the middle of a sandwich, with every level equally sovereign with respect to the global stability of the organism.’ (Yates, 1993, p. 212).

These warnings eventually formed the basis of *The Music of Life* (Noble, 2006), *The Principle of Biological Relativity* (Noble, 2012, 2016) and *Genes and Causation* (Noble, 2008).

## Conclusions

### Evolution biology

The implications for evolutionary biology are profound. I have searched carefully through many textbooks and popular books in that field. I cannot find a single example where the inability of nucleotide sequences in a purely chemical (cell‐free) environment to self‐replicate accurately enough is even acknowledged. Yet the experimental evidence (Deck et al., 2011; Schulman et al., 2012) has been available for over a decade. The best that has been achieved in purely chemical self‐replication is around one error in 10,000 base pairs. Long nucleotide chains (billions of base pairs) cannot therefore replicate simply ‘how crystals are formed’ (Dawkins, 1976, p 17). Yet, that is a *necessary* requirement of both the neo‐Darwinist (Dawkins, 1976, 2024) and Modern Synthesis (Huxley, 1942, 1963) views of evolution. The second edition of Julian Huxley's book, *Evolution, The Modern Synthesis* led to the hardening of the Modern Synthesis view of evolution (Noble & Noble, 2023) and to the mantra that ‘The dogma is a modern version of the Weismann barrier’ (https://simple.wikipedia.org/wiki/Central_dogma_of_molecular_biology). This is a gross error. The one‐way process from nucleotide sequence to amino acid sequence cannot prevent organisms from harnessing stochasticity and producing new DNA sequences when they need to under environmental stress.

A rare exception amongst textbooks on Evolution is *Principles of Evolution, Systems, Species and the History of Life* (Bard, 2017, particularly chapters 12–14). Bard held Waddington's chair at Edinburgh and views evolution from a systems approach. He states that ‘any model that a trait is based on a few trait genes and their interactions is a very weak approximation to reality’. (Bard, 2017, p 233).

Further implications for evolutionary biology can be found in Noble & Noble (2021, 2025).

### Medicine

The implications for medical research relevant to healthcare are clear and are urgent. The focus on genes as DNA sequences has led to an expensive impasse, rather as though we have minutely examined the pixels of a message rather than its meaning. None of Collins’ (1999) predictions have been fulfilled. It is high time to turn to understanding the functional networks. The evolution of different species has led to many ways in which DNA sequences can be shuffled to produce favourable functional outcomes. Different species of high‐altitude birds, for example, have all found ways to enhance the uptake of oxygen to their haemoglobins, but at the genetic level, the processes are virtually random (Natarajan et al., 2015). Humans have also evolved their functional phenotypes from many different ways of rearranging their genes. That is why polygenic scores are not good predictors of disease outcomes (Hingorani et al., 2023). Medical research needs to respect this evolutionary insight.

## Additional information

### Competing interests

There are no competing interests

### Author contributions

The author, D.N., is entirely responsible for all stages of drafting this article.

### Funding

No funding supported this work.

## Supporting information


Peer Review History
