# Supplementary material for: The cardiac pacemakers: A paradigm of robustness in evolutionary biology
Source: J Physiol. 2025 Sep 5;604(13):5159–69. doi: 10.1113/JP287139 (PMC13327764; doi:10.1113/JP287139)
Supplement: Supplementary file 1 — Peer Review History [file TJP-604-5159-s001.pdf]

The Journal of Physiology

The cardiac pacemakers: A paradigm of robustness in evolutionary biology

Denis Noble
DOI: 10.1113/JP287139

Corresponding author(s): Denis Noble (denis.noble@dpag.ox.ac.uk)

The following individual(s) involved in review of this submission have agreed to reveal their identity: Dario DiFrancesco (Referee #1); Edward G Lakatta (Referee #2)

Review Timeline:

Submission Date:	26-Jun-2025
Editorial Decision:	23-Jul-2025
Revision Received:	03-Aug-2025
Accepted:	15-Aug-2025

Senior Editor: Bjorn Knollmann

Reviewing Editor: Axel Loewe

Transaction Report:

(Note: With the exception of the correction of typographical or spelling errors that could be a source of ambiguity, letters and reports are not edited. Depending on transfer agreements, referee reports obtained elsewhere may or may not be included in this compilation. Referee reports are anonymous unless the Referee chooses to sign their reports.)

Dear Professor Noble,

Re: JP-TR-2025-287139 "The cardiac pacemakers: A paradigm of robustness in evolutionary biology" by Denis Noble

Thank you for submitting your manuscript to The Journal of Physiology. It has been assessed by a Reviewing Editor and by 2 expert referees and we are pleased to tell you that it is acceptable for publication following satisfactory revision.

The referee reports are copied at the end of this email.

Please address all the points raised and incorporate all requested revisions or explain in your Response to Referees why a change has not been made. We hope you will find the comments helpful and that you will be able to return your revised manuscript within 4 weeks. If you require longer than this, please contact journal staff: jp@physoc.org.

Your revised manuscript should be submitted online using the link in your Author Tasks: Link Not Available. This link is accessible via your account as Corresponding Author; it is not available to your co-authors. If this presents a problem, please contact journal staff (jp@physoc.org). Image files from the previous version are retained on the system. Please ensure you replace or remove any files that are being revised.

If you do not wish to submit a revised version of your manuscript, you must inform our journal staff (jp@physoc.org) or reply to this email to request withdrawal. Please note that a manuscript must be formally withdrawn from the peer review process at one journal before it may be submitted to another journal.

TRANSPARENT PEER REVIEW POLICY: To improve the transparency of its peer review process, The Journal of Physiology publishes online as supporting information the peer review history of all articles accepted for publication. Readers will have access to decision letters, including Editors' comments and referee reports, for each version of the manuscript, as well as any author responses to peer review comments. Referees can decide whether or not they wish to be named on the peer review history document.

ABSTRACT FIGURES: Authors may use The Journal's premium BioRender account to create/redraw their Abstract Figures (and any other suitable schematic figure). Information on how to access this account is here: https://physoc.onlinelibrary.wiley.com/journal/14697793/biorender-access.

This will enable Authors to create and download high-resolution figures. If authors have used the free BioRender service, they can use the instructions provided in the link above to download a high-resolution version suitable for publication. The link provided should only be used for the purposes of this submission. Authors will be charged for figures created on this account if they are not related to this manuscript submission.

LANGUAGE EDITING AND SUPPORT FOR PUBLICATION: If you would like help with English language editing, or other article preparation support, Wiley Editing Services offers expert help, including English Language Editing, as well as translation, manuscript formatting, and figure formatting at www.wileyauthors.com/eeo/preparation. You can also find resources for Preparing Your Article for general guidance about writing and preparing your manuscript at www.wileyauthors.com/eeo/prepresources.

REVISION CHECKLIST: Upload a full Response to Referees file. To create your 'Response to Referees' copy all the reports, including any comments from the Senior and Reviewing Editors, into a Microsoft Word, or similar, file and respond to each point, using font or background colour to distinguish comments and responses and upload as the required file type.

Please upload two versions of your manuscript text: one with all relevant changes highlighted and one clean version with no changes tracked. The manuscript file should include all tables and figure legends, but each figure/graph should be uploaded as separate, high-resolution files.

You may also upload:

- 'Potential Cover Art' for consideration as the issue's cover image
- Appropriate Supporting Information (Video, audio or data set: see https://jp.msubmit.net/cgi-bin/main.plex?form_type=display_requirements#supp).

We look forward to receiving your revised submission.

If you have any queries, please reply to this email and we will be pleased to advise.

Yours sincerely,

Bjorn Knollmann
Senior Editor
The Journal of Physiology

REQUIRED ITEMS

- Please include an Abstract Figure file, as well as the Figure Legend text within the main article file. The Abstract Figure is a piece of artwork designed to give readers an immediate understanding of the Review Article and should summarise the main conclusions. If possible, the image should be easily 'readable' from left to right or top to bottom. It should show the physiological relevance of the Review so readers can assess the importance and content of the article. Abstract Figures should not merely recapitulate other figures in the Review. Please try to keep the diagram as simple as possible and without superfluous information that may distract from the main conclusion of the Review. Abstract Figures must be provided by authors no later than the revised manuscript stage and should be uploaded as a separate file during online submission labelled as File Type 'Abstract Figure'. Please ensure that you include the figure legend in the main article file. All Abstract Figures will be sent to a professional illustrator for redrawing and you may be asked to approve the redrawn figure before your paper is accepted.

- Your MS must include a complete "Additional information section" with the following 4 headings and content:

Competing Interests: A statement regarding competing interests. If there are no competing interests, a statement to this effect must be included. All authors should disclose any conflict of interest in accordance with journal policy.

Author contributions: Each author should take responsibility for a particular section of the study and have contributed to writing the paper. Acquisition of funding, administrative support or the collection of data alone does not justify authorship; these contributions to the study should be listed in the Acknowledgements. Additional information such as 'X and Y have contributed equally to this work' may be added as a footnote on the title page.

It must be stated that all authors approved the final version of the manuscript and that all persons designated as authors qualify for authorship, and all those who qualify for authorship are listed.

Funding: Authors must indicate all sources of funding, including grant numbers. If authors have not received funding, this must be stated.

It is the responsibility of authors funded by RCUK to adhere to their policy regarding funding sources and underlying research material. The policy requires funding information to be included within the acknowledgement section of a paper. Guidance on how to acknowledge funding information is provided by the Research Information Network. The policy also requires all research papers, if applicable, to include a statement on how any underlying research materials, such as data, samples or models, can be accessed. However, the policy does not require that the data must be made open. If there are considered to be good or compelling reasons to protect access to the data, for example commercial confidentiality or legitimate sensitivities around data derived from potentially identifiable human participants, these should be included in the statement.

Acknowledgements: Acknowledgements should be the minimum consistent with courtesy. The wording of acknowledgements of scientific assistance or advice must have been seen and approved by the persons concerned. This section should not include details of funding.

- Please upload separate high quality figure files via the submission form.

- Author profile(s) must be uploaded via the submission form. Authors should submit a short biography (no more than 100 words for one author or 150 words in total for two authors) and a portrait photograph of the two leading authors on the paper. These should be uploaded and clearly labelled together in a Word document with the revised version of the manuscript. Any standard image format for the photograph is acceptable, but the resolution should be at least 300 DPI and preferably more. A group photograph of all authors is also acceptable, providing the biography for the whole group does not exceed 150 words.

- It is the authors' responsibility to obtain any necessary permissions to reproduce previously published material and to list these within the main article file. For information, please see: https://jp.msubmit.net/cgi-bin/main.plex?

form_type=display_requirements#permissions.

EDITOR COMMENTS

Reviewing Editor:

Your review was assessed by two expert referees who both point out that it is a valuable and potentially impactful contribution to the field.

To consider it for publication in The Journal of Physiology, please address the points mentioned in the referee reports:

- A more comprehensive overview of redundancy mechanisms underlying cardiac pacemaking. For most of these mechanisms, a short mention and reference to other, recent review papers might suffice.

- Inclusion of additional references including those pointed out by referee #1 but also including some more recent relevant articles for the topic of this review.

Senior Editor:

I concur with the reviewing editor's assessment.

REFEREE COMMENTS

Referee #1:

This is an excellent review covering decades of the author's activity across a range of research fields that extend from Cardiac Electrophysiology to Evolutionary Biology, which the author has always mastered through the proposal of provocative new ideas and landmark discoveries.

After revising the fundamentals of biology and the standard theories that drive most clinically relevant studies of our days, the author illustrates the reasons why the gene-centric approach applied to clinical research, on which typically Genome-Wide Association Studies are based, often fails to determine the cause, or causes, of a disease.

Even more importantly, he proposes ways by which a different approach based on physiological experimentation and modelling can, by studying "causation" rather than "association", lead to more clinically useful results. The proposal of a new role for Physiology, in its approach to address "causation" rather than "association" in biological processes, is in my view among the most important and provocative ideas put forward by the author in this review and his long-standing work.

To illustrate how a physiology-based approach is better suited to distinguish between causation and association than a genomic approach, the author explains the concept of "robustness" in the control of physiological processes by complex "functional" networks that interact not only with the standard DNA-RNA-protein scheme, but also importantly with the environment. In this perspective DNA clearly appears therefore to be just one of the actors in a quite complex biological networking.

Functional networks give a new vision of how biological processes are controlled. and provide a framework for the understanding of how "robustness" in biological functions is generated.

As a paradigm of robustness, the author selects data from early work on the cardiac pacemaker mechanism. Several processes, such as ionic currents and Calcium ion handling mechanisms, contribute to cardiac pacemaking. All these processes are finely tuned together, and changes in any of these processes has limited effects on the ability to generate rhythm, although details, such as pacemaking rate, can vary. In 1992, by a combination of electrophysiological experiments and numerical modelling, the author and coworkers showed how pacemaker rhythm could be maintained even if one of the contributing factors, i.e. the Na background current, was varied over a large window. This was shown to be caused by a mechanism whereby changes in the background Na current INaB automatically generate changes in the funny current If that oppose to the original ones, in such a way as to stabilize the pacemaker function.

Starting from simple electrophysiological measurements and numerical modelling from early studies of cardiac pacemakers and the "robustness" of cardiac pacemaking, this review provides a concise account of how the concept of robustness can be explained based on the complexity of functional networks. This demands a shift in the strategy in clinical research towards approaches that can distinguish between causation and association. Physiology can achieve this, while Genome sequencing alone cannot. This is a very important message and a major strength of the review.

Special

I do not have special comments, except to note that some quotations refer to a new book by the author (Noble, 2025), which is still not available and will be published this autumn. Figure 4 is also from that source. The same source is also quoted in relation to the story of how the decision to modify the Hodgkin-Huxley (1952) nerve equation led to the author's 1960 and 1962 papers, and the many revisions that occurred in the interpretation and description of pacemaker theories in the three following decades until the Noble et al paper in 1992.

Considering the significance of that period-marked by revolutionary discoveries that led to a complete revision of the previous pacemaker theory-and considering that the author has himself published several reviews covering this topic (among which a well-cited Annual Review in 1984, J. Phyiol. 353, 1-50), I wonder if it wouldn't be helpful to include this and maybe other key citations to ensure they are immediately accessible to the reader.

Referee #2:

Summary/General Comments

Noble's short review presents his personal view on the present state of biology and highlights the importance of physiological studies over genetics. One of his major points is that while significant strides have been made in understanding the role of genes in biological function, genetics, especially in isolation, still faces limitations in providing a complete picture of complex biological processes. In general, Noble argues that the physiological functions are robust, due to numerous redundant mechanisms, whereas genetic data, e.g. GWAS, provide only association (at best) with functions or diseases, but cannot uncover the complex and redundant mechanisms involved in biophysical processes (like ion currents) occurring within and among cells and tissues. Hence, physiology is needed to figure out the actual mechanisms of pathology, and the demise of physiology in favor of -omics is deplorable.

Noble seems to express bitter resentment that many physiology departments in the UK are closed, whereas implications for evolutionary biology and for medical research are profound. He states that reliance on genomics has led to a gene-centric impasse in health care, which requires a shift in favor of physiological studies that can reveal genuine causation rather than just association. Specifically, he argues that DNA is not a good self-replicator in very large genomes, and that cells implement specific mechanisms to correct errors. As he notes, "The error rate would amount to hundreds of thousands in a genome of 3 billion base pairs. Living cells can orchestrate the enzymes necessary for nearly perfect replication before cell division by correcting those errors." Thus, as key descriptors of his review, Noble reduces The Central Dogma of Molecular Biology to "be viewed simply as a central chemical process for the production of proteins", but that physiology determines the true (redundant) mechanisms and robust functions, i.e. "causation in physiological networks".

Noble next illustrates his view using an example of robust pacemaker function, which he studied for many years, and to which he has made a number of seminal contributions. He refers to his development of the first mechanistic numerical

model of the cardiac action potential (in 1960), following the Hodgkin-Huxley approach. Later, to his "surprise", the "funny" current (If) was discovered and introduced into the formulations of his pacemaker cell model. In this review Noble describes If to be a major redundant mechanism that is more activated at slower AP rates, stabilizing the slow AP firing rate.

Specific Comments

The arguments made by the author are reasonable. Indeed, while genetics and molecular biology have "stolen the show" in biological sciences, their progress in our understanding and treatment of many diseases falls short of our expectations. However, the paper provides just a specific slice of the problem focusing on gene replication and redundancies. A danger and limitation of such an opinion is that it can lead to the extreme reductionist view that physiology alone can solve all problems. The author's premise seems to be that the universe is divided into two parts, i.e., Central Dogma of Molecular Biology and Physiology. This is way too simple and also unrealistic. As has been pointed out by others, reality reflects constant interactions of DNA and its environments (nuclear, cytosolic, tissue, organ, organism, an organism's behavior (lifestyle) and physiology, other organisms, society, terra-firma, atmosphere, and cosmic environments) that occur in the context of the passage of time. In sum, the position put forth in this review does not seem take into account the fact that a time constraint cannot be placed on the rate at which scientific discoveries emerge and that the major investment in science is time. Thus, pointing out shortcomings of comments made by Francis Collins in 1999 seems to be naive to the power of the passage of time in the process of scientific discovery.

Shifting to physiology, the examples provided by Noble regarding redundancies within pacemaker cells are largely only given with respect to membrane ion currents in a single pacemaker cell. Other redundant mechanisms at the frontier of pacemaker systems research are not sufficiently described or not even mentioned, e.g. structural redundancies (e.g. iSAN and sSAN, multiple SAN exits), diverse cell populations (e.g. cells resistant to acetylcholine, latent pacemakers), calcium oscillator, biochemical drivers (cAMP production and phosphorylation), stretch-activated mechanisms, etc. Thus, the point about redundancies within cardiac pacemaker physiology could be stronger, more interesting, and more up-to date if some of these redundant mechanisms were to be discussed in the piece.

END OF COMMENTS

REQUIRED ITEMS

- Please include an Abstract Figure file, as well as the Figure Legend text within the main article file. The Abstract Figure is a piece of artwork designed to give readers an immediate understanding of the Review Article and should summarise the main conclusions. If possible, the image should be easily 'readable' from left to right or top to bottom. It should show the physiological relevance of the Review so readers can assess the importance and content of the article. Abstract Figures should not merely recapitulate other figures in the Review. Please try to keep the diagram as simple as possible and without superfluous information that may distract from the main conclusion of the Review. Abstract Figures must be provided by authors no later than the revised manuscript stage and should be uploaded as a separate file during online submission labelled as File Type 'Abstract Figure'. Please ensure that you include the figure legend in the main article file. All Abstract Figures will be sent to a professional illustrator for redrawing and you may be asked to approve the redrawn figure before your paper is accepted.

I have constructed the simplest possible abstract figure to illustrate the main argument and conclusions of the paper.

- Your MS must include a complete "Additional information section" with the following 4 headings and content:

Competing Interests: A statement regarding competing interests. If there are no competing interests, a statement to this effect must be included. All authors should disclose any conflict of interest in accordance with journal policy.

Competing interests. There are no competing interests

Author contributions: Each author should take responsibility for a particular section of the study and have contributed to writing the paper. Acquisition of funding, administrative support or the collection of data alone does not justify authorship; these contributions to the study should be listed in the Acknowledgements. Additional information such as 'X and Y have contributed equally to this work' may be added as a footnote on the title page.

Author Contributions. The author, D Noble, is entirely responsible for all stages of drafting this article.

It must be stated that all authors approved the final version of the manuscript and that all persons designated as authors qualify for authorship, and all those who qualify for authorship are listed.

Funding: Authors must indicate all sources of funding, including grant numbers. If authors have not received funding, this must be stated.

Funding. No funding supported this work.

It is the responsibility of authors funded by RCUK to adhere to their policy regarding funding sources and underlying research material. The policy requires funding information to be included within the acknowledgement section of a paper. Guidance on how to acknowledge funding information is provided by the Research Information Network. The policy also requires all research papers, if applicable, to include a statement on how any underlying research materials, such as data, samples or models, can be accessed. However, the policy does not require that the data must be made open. If there are considered to be good or compelling reasons to protect access to the data, for example commercial confidentiality or legitimate sensitivities around data derived from potentially identifiable human participants, these should be included in the statement.

Acknowledgements: Acknowledgements should be the minimum consistent with courtesy. The wording of acknowledgements of scientific assistance or advice must have been seen and approved by the persons concerned. This section should not include details of funding.

Acknowledgements. I acknowledge valuable comments by Reine Bourret

- Please upload separate high quality figure files via the submission form.

Those will be provided with the resubmission

- Author profile(s) must be uploaded via the submission form. Authors should submit a short biography (no more than 100 words for one author or 150 words in total for two authors) and a portrait photograph of the two leading authors on the paper. These should be uploaded and clearly labelled together in a Word document with the revised version of the manuscript. Any standard image format for the photograph is acceptable, but the resolution should be at least 300 DPI and preferably more. A group photograph of all authors is also acceptable, providing the biography for the whole group does not exceed 150 words.

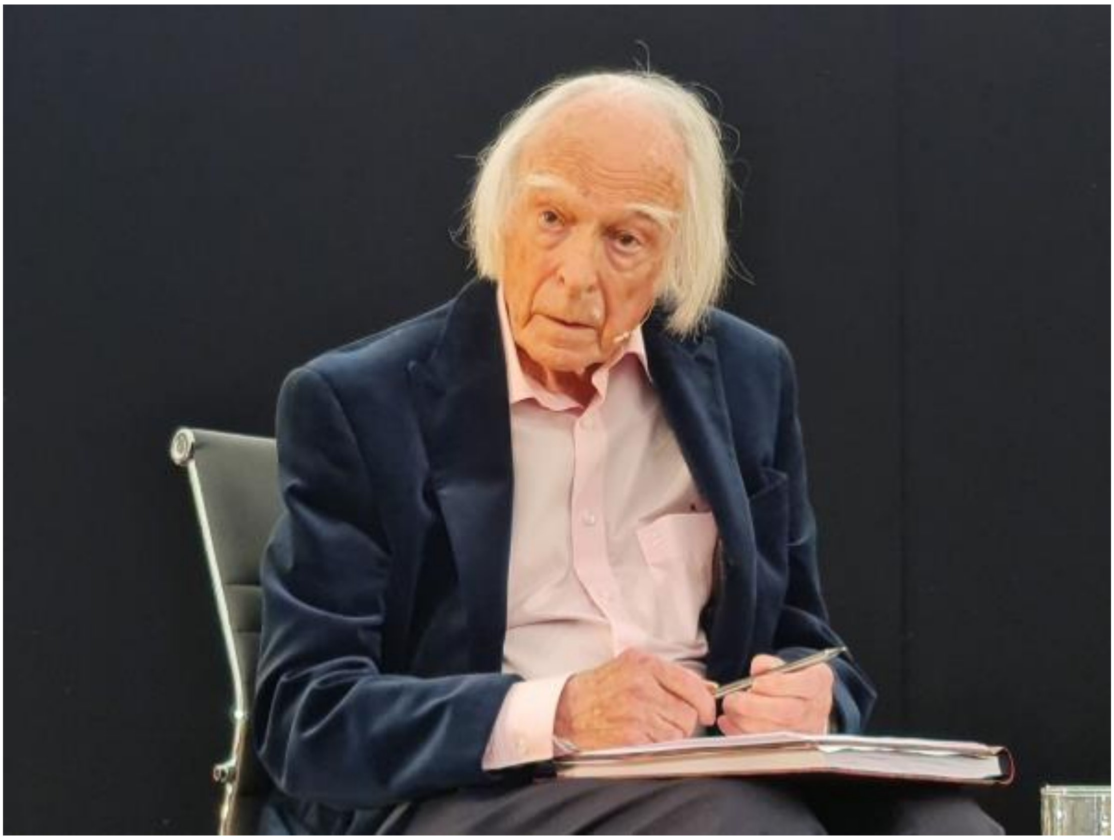

-

Denis Noble debating with Richard Dawkins in 2022

Denis Noble is Emeritus Professor of Cardiovascular Physiology at the University of Oxford. He was the first to create successful mathematical models of heart pacemaker activity in 1960/1962 and has been active in this field subsequently, leading to understanding the robust nature of heart rhythm. He received the Lomonosov Grand Gold Medal of the Russian Academy of Sciences for this work in 2022. He has been

successively Secretary of The Physiological Society, Secretary-General and later President of the International Union of Physiological Sciences. He is an outspoken critic of neo-Darwinist and Modern Synthesis interpretations of Evolutionary Biology.

- It is the authors' responsibility to obtain any necessary permissions to reproduce previously published material and to list these within the main article file. For information, please see: https://jp.msubmit.net/cgi-bin/main.plex?form_type=display_requirements#permissions.

All the images are from my own work, except for Figure 1, which is available on wikipedia.

EDITOR COMMENTS

Reviewing Editor:

Your review was assessed by two expert referees who both point out that it is a valuable and potentially impactful contribution to the field.

To consider it for publication in The Journal of Physiology, please address the points mentioned in the referee reports:

- A more comprehensive overview of redundancy mechanisms underlying cardiac pacemaking. For most of these mechanisms, a short mention and reference to other, recent review papers might suffice.

- Inclusion of additional references including those pointed out by referee #1 but also including some more recent relevant articles for the topic of this review.

Senior Editor:

I concur with the reviewing editor's assessment.

REFEREE COMMENTS

Referee #1:

This is an excellent review covering decades of the author's activity across a range of research fields that extend from Cardiac Electrophysiology to Evolutionary Biology, which the author has always mastered through the proposal of provocative new ideas and landmark discoveries.

After revising the fundamentals of biology and the standard theories that drive most clinically relevant studies of our days, the author illustrates the reasons why the gene-centric approach applied to clinical research, on which typically Genome-Wide Association Studies are based, often fails to determine the cause, or causes, of a disease.

Even more importantly, he proposes ways by which a different approach based on physiological experimentation and modelling can, by studying "causation" rather than "association", lead to more clinically useful results. The proposal of a new role for Physiology, in its approach to address "causation" rather than "association" in biological processes, is in my view among the most important and provocative ideas put forward by the author in this review and his long-standing work.

To illustrate how a physiology-based approach is better suited to distinguish between causation and association than a genomic approach, the author explains the concept of "robustness" in the control of physiological processes by complex "functional" networks that interact not only with the standard DNA-RNA-protein scheme, but also importantly with the environment. In this perspective DNA clearly appears therefore to be just one of the actors in a quite complex biological networking.

Functional networks give a new vision of how biological processes are controlled. and provide a framework for the understanding of how "robustness" in biological functions is generated.

As a paradigm of robustness, the author selects data from early work on the cardiac pacemaker mechanism. Several processes, such as ionic currents and Calcium ion handling mechanisms, contribute to cardiac pacemaking. All these processes are finely tuned together, and changes in any of these processes has limited effects on the ability to generate rhythm, although details, such as pacemaking rate, can vary. In 1992, by a combination of electrophysiological experiments and numerical modelling, the author and coworkers showed how pacemaker rhythm could be maintained even if one of the contributing factors, i.e. the Na background current, was varied over a large window. This was shown to be caused by a mechanism whereby changes in the background Na current INaB automatically generate changes in the funny current If that oppose to the original ones, in such a way as to stabilize the pacemaker function.

Starting from simple electrophysiological measurements and numerical modelling from early studies of cardiac pacemakers and the "robustness" of cardiac pacemaking, this review provides a concise account of how the concept of robustness can be explained based on the complexity of functional networks. This demands a shift in the strategy in clinical research towards approaches that can distinguish between causation and association. Physiology can achieve this, while Genome sequencing alone cannot. This is a very important message and a major strength of the review.

I thank the referee for these comments, which accurately summarize what I am doing in this article.

Special

I do not have special comments, except to note that some quotations refer to a new book by the author (Noble, 2025), which is still not available and will be published this autumn. Figure 4 is also from that source.

The 2025 book, *The Pacemaker Channels of the Heart* is now listed on Amazon, can already be ordered, and will be published next month.

The same source is also quoted in relation to the story of how the decision to modify the Hodgkin-Huxley (1952) nerve equation led to the author's 1960 and 1962 papers, and the many revisions that occurred in the interpretation and description of pacemaker theories in the three following decades until the Noble et al paper in 1992.

Considering the significance of that period-marked by revolutionary discoveries that led to a complete revision of the previous pacemaker theory-and considering that the author has himself published several reviews covering this topic (among which a well-cited Annual Review in 1984, J. Phyiol. 353, 1-50), I wonder if it wouldn't be helpful to include this and maybe other key citations to ensure they are immediately accessible to the reader.

Thank you. I have now added reference to the 1984 review. And more references to other work on cardiac functional robustness, as proposed by referee #2

Referee #2:

Summary/General Comments

Noble's short review presents his personal view on the present state of biology and highlights the importance of physiological studies over genetics. One of his major points is that while significant strides have been made in understanding the role of genes in biological function, genetics, especially in isolation, still faces limitations in providing a complete picture of complex biological processes. In general, Noble argues that the physiological functions are robust, due to numerous redundant mechanisms, whereas genetic data, e.g. GWAS, provide only association (at best) with functions or diseases, but cannot uncover the complex and redundant mechanisms involved in biophysical processes (like ion currents) occurring within and among cells and tissues. Hence, physiology is needed to figure out the actual mechanisms of pathology, and the demise of physiology in favor of -omics is deplorable.

Noble seems to express bitter resentment that many physiology departments in the UK are closed, whereas implications for evolutionary biology and for medical research are profound. He states that reliance

on genomics has led to a gene-centric impasse in health care, which requires a shift in favor of physiological studies that can reveal genuine causation rather than just association. Specifically, he argues that DNA is not a good self-replicator in very large genomes, and that cells implement specific mechanisms to correct errors. As he notes, "The error rate would amount to hundreds of thousands in a genome of 3 billion base pairs. Living cells can orchestrate the enzymes necessary for nearly perfect replication before cell division by correcting those errors." Thus, as key descriptors of his review, Noble reduces The Central Dogma of Molecular Biology to "be viewed simply as a central chemical process for the production of proteins", but that physiology determines the true (redundant) mechanisms and robust functions, i.e. "causation in physiological networks".

Noble next illustrates his view using an example of robust pacemaker function, which he studied for many years, and to which he has made a number of seminal contributions. He refers to his development of the first mechanistic numerical model of the cardiac action potential (in 1960), following the Hodgkin-Huxley approach. Later, to his "surprise", the "funny" current (If) was discovered and introduced into the formulations of his pacemaker cell model. In this review Noble describes If to be a major redundant mechanism that is more activated at slower AP rates, stabilizing the slow AP firing rate.

Specific Comments

The arguments made by the author are reasonable. Indeed, while genetics and molecular biology have "stolen the show" in biological sciences, their progress in our understanding and treatment of many diseases falls short of our expectations. However, the paper provides just a specific slice of the problem focusing on gene replication and redundancies. A danger and limitation of such an opinion is that it can lead to the extreme reductionist view that physiology alone can solve all problems.

I am anxious to avoid such an interpretation because it does not represent my view, which is a relativity one: causality is always both ways, up and down. That is the Principle of Biological Relativity developed in *Dance to the Tune of Life* (CUP 2016). As that principle emphasises, “there is no privileged level of causation”.

This statement is reiterated many times in my book *Dance to the Tune of Life, Biological Relativity.* (CUP 2015):

"….there is no 'real' central or privileged level of the body, and no literal bottom or top either" (p 34)
"The principle of Biological Relativity is simply that there is no [privileged level of causation in biology" (p 160)
"'Down' and 'Up' here are metaphors and should be treated carefully. The essential point is the more neutral statement: there is no privileged level of causality" (p 169)

The author's premise seems to be that the universe is divided into two parts, i.e., Central Dogma of Molecular Biology and Physiology.

That is not my view at all, as is evident from the quotes from my book above. I have edited the text of the article to clarify that both are required. That is also what Figure 2 shows.

This is way too simple and also unrealistic. As has been pointed out by others, reality reflects constant interactions of DNA and its environments (nuclear, cytosolic, tissue, organ, organism, an organism's behavior (lifestyle) and physiology, other organisms, society, terra-firma, atmosphere, and cosmic environments) that occur in the context of the passage of time. In sum, the position put forth in this review does not seem take into account the fact that a time constraint cannot be placed on the rate at which scientific discoveries emerge and that the major investment in science is time. Thus, pointing out shortcomings of comments made by Francis Collins in 1999 seems to be naive to the power of the passage of time in the process of scientific discovery.

I think I see what the referee is getting at here. But I don't think my article is just a criticism of Collins' lecture *from the perspective of 2025*. The essence of the argument was already fully presented in my article "Genes and Causation" in Phil Trans Roy Soc in 2008. And, even well before Collins' 1999 lecture, in 1993 I co-edited and contributed to *The Logic of Life: The challenge of integrative physiology* (OUP, Eds Boyd & Noble). Many of the arguments in that book, such as my criticisms of gene-centric biology as presented by Richard Dawkins, and the articles in that volume by EM Southern, on "Physiology and Genes", and by Stephen J Gould (also a major critic of gene-centric biology) on

“Evolution of Organisms”, made all the main points clear *well before 1999*.

Significantly from my perspective, that book followed on quite quickly after the 1992 article that forms the experimental basis of the current review. Had the genomics community taken any notice of what physiologists like me were saying and writing in the 1990s some of the hype could have been avoided.

I have now cited 3 articles from *The Logic of Life* in a new section **When were the alarm bells ringing?** And how that developed into *The Music of Life*, The Principle of Biological Relativity, and “Genes and Causation”.

Shifting to physiology, the examples provided by Noble regarding redundancies within pacemaker cells are largely only given with respect to membrane ion currents in a single pacemaker cell.

Most of the work on the reciprocal interaction between different processes in pacemaker rhythm were performed *before* 1992 on *multicellular node preparations*, not on single cells. The single cell results provide the most accurate fitting of equations to experimental data, which is why I have used those results in this review. But the conclusions apply to more than single cell work.

Other redundant mechanisms at the frontier of pacemaker systems research are not sufficiently described or not even mentioned, e.g. structural redundancies (e.g. iSAN and sSAN, multiple SAN exits), diverse cell populations (e.g. cells resistant to acetylcholine, latent pacemakers), calcium oscillator, biochemical drivers (cAMP production and phosphorylation), stretch-activated mechanisms, etc. Thus, the point about redundancies within cardiac pacemaker physiology could be stronger, more interesting, and more up-to date if some of these redundant mechanisms were to be discussed in the piece.

I thank the referee for pointing this out. I have added further references as suggested.

My main focus in this review is to establish the wide application of the genetic buffering (redundancy) argument *to biology as a whole*. Hence the importance of Hillenmeyer et al’s 2008 work on yeast, and the explanation of the widespread observation by many authors now that

most GWAS have revealed very low association scores. My intention is to generalise the conclusions to go *way beyond the heart pacemaker* to consider their applications to biology as a whole. My review was not therefore intended to be a review of the heart pacemaker mechanisms in general. The mechanisms I chose to illustrate the strong redundancy, and therefore the difference between association and causation, achieve just that. No more.

Dear Professor Noble,

Re: JP-TR-2025-287139R1 "The cardiac pacemakers: A paradigm of robustness in evolutionary biology" by Denis Noble

We are pleased to tell you that your paper has been accepted for publication in The Journal of Physiology.

Please see Editor Comments below for a tiny amendment to be made at proof stage.*

TRANSPARENT PEER REVIEW POLICY: To improve the transparency of its peer review process, The Journal of Physiology publishes online as supporting information the peer review history of all articles accepted for publication. Readers will have access to decision letters, including Editors' comments and referee reports, for each version of the manuscript, as well as any author responses to peer review comments. Referees can decide whether or not they wish to be named on the peer review history document.

The last Word (or similar) version of the manuscript provided will be used by the Production Editor to prepare your proof. When this is ready you will receive an email containing a link to Wiley's Online Proofing System. The proof should be thoroughly checked and corrected as promptly as possible.

Authors should note that it is too late at this point to offer corrections prior to proofing. Major corrections at proof stage, such as changes to figures, will be referred to the Editors for approval before they can be incorporated. Only minor changes, such as to style and consistency, should be made at proof stage. Changes that need to be made after proof stage will usually require a formal correction notice.

All queries at proof stage should be sent to: TJP@wiley.com.

Are you on Twitter? Once your paper is online, why not share your achievement with your followers? Please tag The Journal (@jphysiol) in any tweets and we will share your accepted paper with our 30,000 followers!

Yours sincerely,

Bjorn Knollmann
Senior Editor
The Journal of Physiology

P.S. - You can help your research get the attention it deserves! Check out Wiley's free Promotion Guide for best-practice recommendations for promoting your work at www.wileyauthors.com/eeo/guide. You can learn more about Wiley Editing Services which offers professional video, design, and writing services to create shareable video abstracts, infographics, conference posters, lay summaries, and research news stories for your research at www.wileyauthors.com/eeo/promotion.

IMPORTANT NOTICE ABOUT OPEN ACCESS: To assist authors whose funding agencies mandate public access to published research findings sooner than 12 months after publication, The Journal of Physiology allows authors to pay an Open Access (OA) fee to have their papers made freely available immediately on publication.

The Corresponding Author will receive an email from Wiley with details on how to register or log-in to Wiley Authors Services where you will be able to place an order.

You can check if your funder or institution has a Wiley Open Access Account here: https://authorservices.wiley.com/author-resources/Journal-Authors/licensing-and-open-access/open-access/author-compliance-tool.html.

EDITOR COMMENTS

Reviewing Editor:

Your manuscript was assessed again and both expert referees confirmed that all of their concerns have been addressed sufficiently.

Senior Editor:

Thank you for your excellent contribution. At proof stage, please remember to quote your 1984 J. Physiol. review paper in

the text. It is only listed in the references.

REFEREE COMMENTS

Referee #1:

Thank you for your response, I do not have further comments.

Please remember to quote your 1984 J. Physiol. review paper in the text. You have added the paper to the list of References as suggested, but I do not see it has been cited in the text.

Referee #2:

Thank you for addressing our comments.
